# Phylogeographic analyses of the pampas cat (*Leopardus colocola*; Carnivora, Felidae) reveal a complex demographic history

**DOI:** 10.1590/1678-4685-GMB-2017-0079

**Published:** 2018

**Authors:** Anelisie da Silva Santos, Tatiane Campos Trigo, Tadeu Gomes de Oliveira, Leandro Silveira, Eduardo Eizirik

**Affiliations:** 1 Laboratório de Biologia Genômica e Molecular, Escola de Ciências, Pontifícia Universidade Católica do Rio Grande do Sul (PUCRS), Porto Alegre, RS, Brazil; 2 Setor de Mastozoologia, Museu de Ciências Naturais, Fundação Zoobotânica do Rio Grande do Sul. Porto Alegre, RS, Brazil; 3 Universidade Estadual do Maranhão (UEMA), São Luís, MA, Brazil; 4 Instituto Pró-Carnívoros, Atibaia, SP, Brazil; 5 Instituto Onça-Pintada, Mineiros, GO, Brazil

**Keywords:** Phylogeography, population genetics, mitochondrial DNA, conservation genetics, historical demography

## Abstract

The pampas cat is a small felid that occurs in open habitats throughout much of South America. Previous studies have revealed intriguing patterns of morphological differentiation and genetic structure among its populations, as well as molecular evidence for hybridization with the closely related *L. tigrinus*. Here we report phylogeographic analyses encompassing most of its distribution (focusing particularly on Brazilian specimens, which had been poorly sampled in previous studies), using a novel dataset comprising 2,143 bp of the mitogenome, along with previously reported mtDNA sequences. Our data revealed strong population strutucture and supported a west-to-east colonization process in this species’ history. We detected two population expansion events, one older (*ca.* 200 thousand years ago [kya]) in western South America and another more recent (*ca.* 60-50 kya) in eastern areas, coinciding with the expansion of savanna environments in Brazil. Analyses including *L. tigrinus* individuals bearing introgressed mtDNA from *L. colocola* showed a complete lack of shared haplotypes between species, indicating that their hybridization was ancient. Finally, we observed a close relationship between Brazilian/Uruguayan *L. colocola* haplotypes and those sampled in *L. tigrinus*, indicating that their hybridization was likely related to the demographic expansion of *L. colocola* into eastern South America.

## Introduction

The formation of the Panamanian Isthmus led to the colonization of South America by several lineages of North American mammals, some of which gave rise to endemic Neotropical adaptive radiations (Eizirik, 2012). This is the case of the genus *Leopardus* (Mammalia, Carnivora, Felidae), composed by at least eight species of small and medium-sized cats that occur in a variety of habitats across the Neotropics, and whose diversification began 3 to 5 million years ago (MYA) (Nowell and Jackson, 1996; Eisenberg and Redford, 1999; Johnson *et al.*, 2006; Trigo *et al.*, 2013; Li *et al.*, 2016).

**Figure 1:**
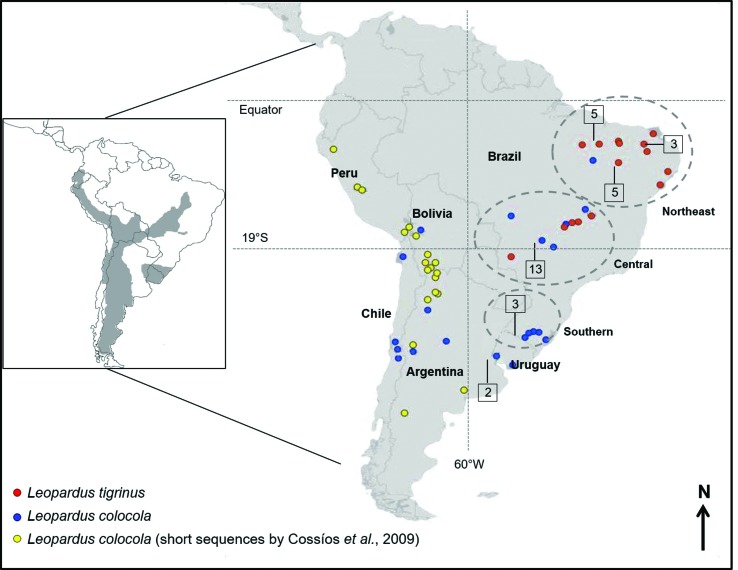
Geographic distribution of *Leopardus colocola* and *L. tigrinus* samples analyzed in this study. Dotted circles indicate the Brazilian regions considered in the analyses. The numbers in squares indicate the number of samples belonging to the same geographic region. The blue and red circles represent samples with mitochondrial sequences generated by this study, while yellow circles represent the geographic origin of haplotypes reported by Cossíos *et al.* (2009). The inset map on the left shows the geographic distribution of *L. colocola* (dark grey) [modified from the IUCN (2011) *IUCN Red List of Threatened Species*, http://www.iucnredlist.org].

The pampas cat (*Leopardus colocola*) is considered one of the least known species of this genus (Silveira 1995; Nowell and Jackson, 1996). It presents an extensive geographic distribution (Figure 1), occurring from Ecuador (or perhaps southwestern Colombia) to the Strait of Magellan. It is mainly associated with open habitats, such as the Argentinean and Uruguayan pampas, Bolivian and Paraguayan Chaco and the high altitude fields along the Andean mountain chain, but may also be found in forested habitats. In Brazil, it is restricted to open habitats such as the Pampas biome in southern Brazil and the Cerrado and Pantanal biomes in the central and northeastern parts of the country (Silveira, 1995; Nowell and Jackson, 1996; Eisenberg and Redford, 1999; Pereira *et al.*, 2002; Ruiz-Garcia *et al.*, 2003; Villalba and Delgado, 2005; Godoi *et al.*, 2010, Queirolo *et al.*, 2013, Lucherini *et al.*, 2016). The pampas cat is considered Near Threatened worldwide, but its distinctive evolutionary units (see below) can all be considered as Vulnerable individually (Queirolo *et al.*, 2013, Lucherini *et al.*, 2016), or perhaps even more severely threatened, pending on regional in-depth analyses.

Substantial morphological variation has been observed across pampas cat regional populations, leading to considerable controversy regarding its species-level taxonomic status (see Kitchener *et al*. [2017] for a recent review). Garcia-Perea (1994), for example, based on cranial morphology and coat patterns, suggested the subdivision of the pampas cat into three distinct species (*L. colocolo*, *L. braccatus* and *L. pajeros*), each containing subspecies-level units. A subsequent molecular study based on three mitochondrial genes (16S rDNA, *ATP8* and *ND5*) indicated strong genetic structuring among different pampas cat populations (Johnson *et al.*, 1999), but did not support the species-level subdivision proposed by Garcia-Perea (1994), given that the observed phylogroups were rather recently diverged. Nevertheless, a subsequent systematic treatise (Wozencraft, 2005) recognized the three species proposed by Garcia-Perea (1994), and a more recent morphological analysis (Nascimento FO, 2010, Doctoral thesis, Universidade de São Paulo, São Paulo) further proposed the subdivision of the complex into six distinct species.

The existence of highly structured populations in this cat species has been supported by recent molecular studies (Napolitano *et al.*, 2008; Cossíos *et al.*, 2009), although not always matching the proposed morphological partitions. The study of Napolitano *et al.* (2008) was concentrated in areas of northern Chile, where the authors found a lack of haplotype sharing with neighbouring geographic areas, supporting the hypothesis that some portions of the pampas cat distribution have experience significant periods of demographic isolation from other regions. The subsequent study by Cossíos *et al.* (2009) reported analyses of mitochondrial genes (*ND5*, control region and *ATP8*) and microsatellie loci for a data set focused on the central Andes. These authors found strong genetic differentiation among several regional populations, supporting the recognition of at least four Management Units (MU) for conservation purposes.

In spite of the advances provided by these genetic analyses, the evolutionary history of the pampas cat remains incompletely understood, mainly because these studies have focused on partial sampling of its geographic distribution. Populations of central and southern Brazil, for example, were poorly represented (or unrepresented) in these earlier investigations, and thus their evolutionary relationships with those from western South America remain obscure.

Interestingly, Brazilian populations of *L. colocola* present an additional layer of evolutionary complexity, as we have documented that they underwent a massive historical process of hibridization and unidirectional introgression affecting a congeneric species, *Leopardus tigrinus* (Trigo *et al.*, 2013). Remarkably, this process has led to a complete replacement of the *L. tigrinus* mitochondrial genome by that originating from *L. colocola*, with no evidence of this historical admixture having been so far detected in any nuclear marker (Trigo *et al.*, 2013). This uncommon pattern is likely a consequence of ancient episodes of hybridization, probably involving primary matings between *L. colocola* females and *L. tigrinus* males, followed by backcrossing of female hybrids to male *L. tigrinus* for multiple generations. Such crosses are expected to dilute the signal of introgression in the nuclear genome due to several generations of cumulative backcrossing, explaining the genetic pattern we have observed.

In addition, according to Trigo *et al.* (2013), *L. tigrinus* was found to be mainly associated to two Brazilian Biomes, Cerrado and Caatinga, which tend to present open/dry vegetation types that are also the typical habitats used by *L. colocola* in Brazil, in stark contrast to the Atlantic Forest associated with *L. guttulus.* This observation led us to hypothesize that its ancient hybridization with *L. colocola* might have been involved with adaptation of *L. tigrinus* to such open biomes. Such observations suggest an intriguing evolutionary process that still requires further investigation, including the estimation of its temporal, spatial and demographic contexts. At this time, the only genetic system that allows an assessment of these issues is the mitochondrial DNA (mtDNA), since it so far holds the only available record of this ancient episode of hybridization.

In this context, the main goal of this present study was to investigate the evolutionary history of *L. colocola* based on the analysis of mtDNA segments, aiming to characterize its phylogeographic patterns and demographic history, as well as to gain additional insights into its hybridization/introgression event with *L. tigrinus.* In particular, we pursued the following specific objectives: 1) to assess the genetic relationships between western *L. colocola* populations and those from eastern South America (Brazil and Uruguay); 2) to estimate the geographic origin and age of the mitochondrial DNA haplotypes introgressed into *L. tigrinus,* and 3) to analyze the correlation between the genetically identified groups and the morphology-based taxa proposed by Garcia-Perea (1994). Clarifying these issues is relevant not only from an evolutionary biology and taxonomy standpoint, but should also have significant impacts on conservation and management strategies on behalf of this species.

## Materials and Methods

### Sample collection

We generated mtDNA sequence data from 40 *L. colocola* individuals from Brazil, Argentina, Uruguay, Chile and Bolivia, as well as 28 *L. tigrinus* from central and northeastern Brazil (see Figure 1 and Supplemental Table S1). Blood samples were collected from wild animals captured for ecological studies, as well as from captive individuals (preferentially with known geographic origin), and were preserved in a salt saturated solution (100mM Tris, 100mM EDTA, 2% SDS). Tissue samples were obtained from road-killed specimens and maintained in 96% ethanol. Samples of *Leopardus pardalis* (ocelot) and *Leopardus wiedii* (margay) were also included as outgroups in some of the analyses.

### DNA extraction, amplification and sequencing

DNA extraction was performed using a standard phenol/chloroform protocol (Sambrook *et al.*, 1989) or using the DNeasy Blood and Tissue Kit (Qiagen, Hilden, Germany) or PureLink^TM^ Genomic DNA Mini Kit (Invitrogen, Carlsbad, CA, USA) following the manufacturers’ instructions. All DNA samples were quantified in a 1% agarose gel stained with GelRed® (Biotium Inc., Fremont, CA, USA) using the LowMass DNA Ladder (Invitrogen).

We amplified four mtDNA segments using the Polymerase Chain Reaction (PCR; Saiki *et al.*, 1985): (I) the 5’ portion of the *ND5* gene, using primers described by Trigo *et al.* (2008); (II) the complete *cytochrome b* gene [*Cytb*] using primers reported by Tchaicka *et al.* (2007) or those described by Irwin *et al.* (1991) and Koepfli and Wayne (1998), which divide the gene into two sub-segments as an alternative approach for use with degraded DNA samples; (III) a segment of the *ATP8* gene using primers described by Johnson *et al.* (1998); and (IV) the last portion of the first hypervariable segment of the mtDNA control region [*CR*], using primers described by Tchaicka *et al.* (2007) and Cossíos *et al.* (2009).

PCR amplifications were performed in a final volume of 20 μL, containing 1X PCR buffer (Invitrogen), 0.2 μM of each primer, 0.2 mM dNTPs, 1.5-2.0 mM MgCl_2_ (Invitrogen), 0.2 U Platinum® Taq Polymerase (Invitrogen) and 5-20 ng of genomic DNA. The addition of 0.2% Triton X-100 to the PCR reaction was used to remove PCR inhibitors in the case of the *ND5*, *Cyt-b* and control region segments. For these three segments, the PCR conditions were identical and began with one step of 94°C for 3 min, 5 cycles (Touchdown) of 94°C for 45 s, 55-51°C for 45 s, 72°C for 1 min 30 s followed by 40 cycles of 94°C for 45 s, 50°C for 30 s, 72°C for 1 min 30 s and final extension of 72°C for 30 min. Thermocycling conditions for the *ATP8* gene consisted of an initial denaturing step at 94°C for 3 min followed by 35 cycles of 94°C for 45 s, 50°C for 45 s, 72°C for 1 min 30 s and final extension of 72°C for 10 min.

PCR products were visualized on a 1% agarose gel stained with GelRed® (Biotium Inc.) and then purified using a protocol based on precipitation with ammonium acetate and isopropanol, or the enzymatic method with the enzymes Exonuclease I (EXO I) and Shrimp Alkaline Phosphatase (SAP). Both strands of each PCR product were sequenced using the DYEnamic ET Dye Terminator Sequencing Kit (GE Healthcare) and analyzed in a MegaBACE 1000 automated sequencer (GE Healthcare). For the mtDNA control region, due to the occurrence of a repetitive region which generates high levels of heteroplasmy (see Eizirik *et al.*, 1998 and Kim *et al.*, 2001 for more examples in other felids), which hampered the stable annealing of the forward primer, only the reverse strand was sequenced. In this case, samples were amplified and sequenced at least twice independently to assess the reproducibility of the observed sequence.

The visualization of electropherograms, as well as the construction of double-strand consensus sequences for each sample, were performed with Geneious Pro 5.5.2 (Drummond *et al.*, 2011). As a complement to data generated in this study, we added sequences previously obtained by Trigo *et al.* (2013) for the *ND5* gene, which include 15 *L. colocola* and 21 *L. tigrinus* individuals (KF679939, KF679949, KF679950, KF679952 - KF679955, KF679958 – KF679961). Shorter sequences for *ATP8, ND5* and control region (133, 270 and 170 base pairs [bp], respectively) reported by Cossíos *et al.* (2009) and covering the Andean portion of the *L. colocola* distribution were also included (FJ648644, FJ648646, FJ648647, FJ648654, FJ648655, FJ648659 – FJ648662, FJ648665, FJ648667, FJ648668, FJ648670, FJ648672, FJ648673, FJ648675, FJ648679, FJ648681, FJ648683) (Figure 1). Novel sequences generated in this study were deposited in GenBank.

### Descriptive analyses and phylogenetic inference

DNA sequences were aligned using the MUSCLE algorithm (Edgar, 2004) implemented in MEGA 5 (Tamura *et al.*, 2011). The alignments were checked and edited manually when necessary. Basic statistics of genetic diversity, including the number of variable sites (V), number of parsimony-informative sites (PI), number of haplotypes (h), nucleotide diversity (π) and haplotype diversity (Hd) were estimated using MEGA 5, ARLEQUIN v.3.5 (Excoffier and Lischer, 2010) and DnaSP 5.0 (Librado and Rozas 2009).

Two data sets were established for use in the analyses described below: (i) Data set A (DSA) with all sequences generated in this study as well as data reported by Trigo *et al.* (2013); and (ii) Data set B (DSB) with all the sequences from DSA, complemented by segments reported by Cossíos *et al.* (2009). The sequences analyzed by Cossíos *et al.* (2009) were shorter in length, so DSB has a broader geographic coverage but a matrix with more missing data.

Phylogenetic analyses were performed with only one representative of each haplotype, and initially employed two different optimality criteria: Bayesian Inference (BI) and Maximum Likelihood (ML). The software jModelTest 0.1.1 (Guindon and Gascuel, 2003; Posada, 2008) was used to determine the best model of nucleotide substitution, applying the Akaike Information Criterion (AIC; Akaike, 1974). For the Bayesian inference, the best model was inferred separately for each of the four mitochondrial segments. We used PhyML 3.0 (Guindon *et al.*, 2010) for the ML analyses, with trees inferred using a heuristic search with a random starting tree, Subtree Pruning Regrafting (SPR) + Nearest-Neighbor-Interchange (NNI), and support estimated by 1,000 bootstrap pseudoreplicates. For initial BI analyses we used MrBayes 3.1 (Ronquist and Huelsenbeck, 2003), including two independent Markov Chain Monte Carlo (MCMC) runs, each containing four Metropolis-coupled chains (one cold and three heated) for five million generations. Trees were sampled every 100 generations, discarding the first 2,500 trees as burn-in. The program Tracer 1.5 (Rambaut and Drummond, 2007) was used for visualization and analyses of MrBayes output files.

Final phylogenetic analyses employed the relaxed Bayesian approached implemented in BEAST 1.6.2 (Drummond and Rambaut, 2007), based on a two-stage strategy. We initially ran the software to estimate the substitution rate of each mitochondrial segment (*ND5*, *Cytb*, *ATP8* and *CR*), using only a few divergent haplotypes and three node-based calibrations. These were based on the credibility intervals estimated previously (Johnson *et al.*, 2006) for three nodes within the genus *Leopardus*: 1) the basal diversification of the genus (2.02 – 4.25 MYA); 2) the divergence between *L. pardalis* and *L. wiedii* (1.01 – 2.41 Mya); and 3) the basal divergence in the clade (*L. jacobita* + *L. colocola* + *L. tigrinus* + *L. geoffroyi* + *L. guigna*) (1.68 – 3.56 Mya). For these runs we used a uniform prior, an uncorrelated lognormal relaxed molecular clock and a Yule prior for the tree, and performed the same analysis separately for the two data sets (DSA and DSB). Trees were linked and the substitution model was set as unlinked, allowing the incorporation of the different substitution models estimated for each segment with jModelTest. We ran BEAST for 50,000,000 steps, sampling every 5,000 iterations, after a discarded burn-in of 50,000 steps. Convergence to the stationary distribution and sufficient sampling were assessed with TRACER. The mean substitution rate (per site per year) obtained in these analyses were as follows: DSA: *ND5* = 2.52 x 10^-8^, *Cytb* = 2.48 x 10^-8^, *ATP8* = 2.93 x 10^-8^, *CR* = 3.53 x 10^-8^; DSB: *ND5* = 2.53 x 10^-8^, *Cytb* = 2.42 x 10^-8^, *ATP8* = 3.87 x 10^-8^, *CR* = 4.89 x 10^-8^.

The second set of BEAST analyses incorporated the mean substitution rates estimated in the first round for each mitochondrial partition, aiming to simultaneously estimate the phylogenetic tree and the TMRCA (the time to the most recent common ancestor) of each pampas cat phylogroup. In this round, we included one copy of all identified haplotypes and assumed a strict clock and a coalescent (constant size) tree prior. The analysis was performed separately for each data set (DSA and DSB) and was run for 100,000,000 steps, sampling every 10,000 iterations, after a discarded burn-in of 100,000 steps, taking into account the stabilization of the traces and the sampled parameters.

### Demographic history and population genetic structure

In addition to the phylogeny-based approaches, haplotype networks were constructed using the median-joining approach (Bandelt *et al.*, 1999) implemented in NETWORK 4.6.0.0 (http://www.fluxus-engineering.com/sharenet.htm) to depict phylogenetic, geographic and potential ancestor-descendent relationships among sequences. In parallel, population structure analyses were performed initially with the software BAPS (Bayesian Analysis of Population Structure) (Corander and Tang, 2007; Corander *et al.*, 2008). We ran the mixture model to assess the most probable number of genetic groups present in our sample, using a range from 1 to 10, and repeated the analysis five times to check for stability.

As a measure of differentiation among geographic groups, we estimated the fixation index Φ_*ST*_ using an Analysis of Molecular Variance (AMOVA) approach (Excoffier *et al.*, 1992) implemented in ARLEQUIN. The correlation between genetic and geographic distances was assessed using a Mantel test (Mantel, 1967) with 100,000 permutations in the program ALLELES IN SPACE (AIS; Miller, 2005).

To obtain estimates of matrilineal gene flow among different geographic populations, we used the coalescent-based Bayesian method implemented in LAMARC (Kuhner, 2006). This software was used to estimate the parameter *theta*, which is the effective population size (Ne) scaled by the mutation rate (μ), along with pairwise migration rates (*M*) for all the defined populations. The number of migrants per generation was obtained by multiplying *M* by *theta* of the recipient population. We performed three independent runs of the Bayesian search strategy, including one long chain of 3,000,000 steps with a sampling increment of 100 (resulting in a total of 30,000 sampled trees), following a burn-in period of 3,000 sampled genealogies. The same analysis was conducted independently for both data sets (DSA and DSB), and the results were visualized in TRACER.

To estimate historical demographic parameters and to evaluate possible scenarios for the colonization of eastern South America by *L. colocola,* we performed Fu’s *F*_s_ neutrality test (Fu, 1997) and Mismatch Distribution Analyses (Rogers and Harpending, 1992) with DnaSP and ARLEQUIN. These tests are used with mtDNA data (assuming selective neutrality) to detect demographic events such as expansions, contractions and bottlenecks (Ramirez-Soriano *et al.*, 2008). Additionally, to estimate possible changes in population size over time, Bayesian Skyline plots were estimated using BEAST. For this purpose, we used the mean substitution rates and their respective 95% credibility intervals estimated by the first round of BEAST analyses (see above) as a normal prior. The MCMC chain was run with 50,000,000 steps, sampling every 5,000 iterations after a discarded burn-in of 50,000 steps.

## Results

### Genetic diversity

We sequenced a total of 2,143 bp of mitochondrial DNA for 40 *L. colocola* and 28 *L. tigrinus* samples (*ND5* = 567 bp, *Cyt-b* = 1028 bp, *ATP8* = 133 bp, *CR* = 415 bp), and designated this alignment as Data set A (DSA). Additonally, we added to these data the shorter sequences reported by Cossíos *et al.* (2009), corresponding to 19 haplotypes, so as to construct Data set B (DSB). The analyses with all segments concatenated led to the resolution of 68 haplotypes for the ingroup (*L. colocola* + *L. tigrinus*) in DSB, 58 of them being sampled only once, and 49 also being represented in DSA (Table 1; see description of haplotypes in Table S1). The concatenated data sets had high haplotypic and low nucleotide diversity, indicating a pattern of rapid and recent diversification. Interestingly, all 19 haplotypes identified in *L. tigrinus* samples were exclusive of that population, with no sharing with *L. colocola* individuals.

**Table 1:** Mitochondrial DNA diversity in *Leopardus colocola* and *L. tigrinus*. Diversity estimates are shown for each data set considered (DSA and DSB), and for each segment independently.

Mitochondrial Segment		*N*	*L* (bp)	*V*	*h*	*H*d (SD)^1^	π (SD)^1^
*ATP8*							
	DSA	64	133	9	10	0,341 (± 0.077)	0.004 (± 0.001)
	DSB	83	133	11	16	0.600 (± 0.063)	0.0109 (± 0.00142)
*ND5*							
	DSA	64	567	33	19	0.859 (± 0.025)	0.005 (± 0.0009)
	DSB	83	271	21	22	0.732 (± 0.048)	0.008 (± 0.001)
*Cytb*							
	DSA	62	1028	33	16	0.878 (± 0.019)	0.00618 (± 0.001)
Control Region							
	DSA	65	415	82	43	0.982 (± 0.007)	0.03475 (± 0.00482)
	DSB	84	171	65	57	0.983 (± 0.006)	0.06965 (± 0.004)
Concatenation							
	DSA	65	2143	124	48	0.985 (± 0.007)	0.01624 (± 0.0018)
	DSB	84	574	97	68	0.9834 (± 0.0059)	0.02776 (± 0.00386)

Notes:

1 Calculation performed with complete deletion and *p-*distance.

Abbreviations: *N* = sequence numbers; *L* = sequence lenght in base pairs (bp); *V* = polymorphic sites; *h* = number of haplotypes; *H*d = haplotypic diversity; π *=* nucleotide diversity; SD = standard deviation.

### Phylogenetic analyses

**Figure 2:**
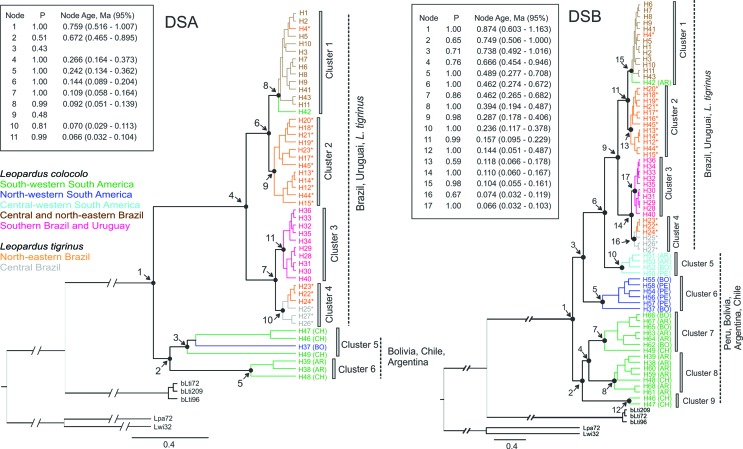
Phylogenetic relationships among *Leopardus colocola* and *L. tigrinus* mitochondrial haplotypes, as assessed with dataset A (DSA) and dataset B (DSB), and depicted in Bayesian trees generated with BEAST. Insets depict each of the main nodes, with posterior probability (P) and node age in million years ago, with respective 95% credibility intervals. The five colors associated to *L. colocola* haplotypes are the same used in Figure 4 to highlight the five genetic groups identified in this species. The grey and orange colors indicate the haplotypes found only in *L. tigrinus* individuals.

All phylogenetic trees constructed with both data sets and both criteria (BI and ML) were congruent with respect to their main topological features. Therefore, only the results of the final analyses, using the relaxed Baysian approach implemented in BEAST, are shown (Figure 2). Haplotypes identified in all *L. colocola* and *L. tigrinus* were grouped into a monophyletic cluster with maximum probability based on both data sets. The TMRCAs estimated for this clade with DSA and DSB were very similar, with a mean ranging from 759 to 874 thousand years ago (kya), and 95% credibility intervals between 516 kya and 1.163 Mya. Three samples from central Brazil were placed in a basal cluster relative to all *L. colocola* and *L. tigrinus* haplotypes, corresponding to two individuals (bLti72 and bLti96) previously recognized as *L. guttulus* by Trigo *et al.* (2013), and one additional sample (bLti209), included in this study. The formation of this cluster, representing *L. guttulus*, supports the inference of a possible area of sympatry between this species and *L. tigrinus* in central Brazil.

Evidence of strong geographic structure was found in both DSA and DSB trees, highlighting the basal position of haplotypes sampled in western regions of South America, and the identification of a well-supported internal group containing all Brazilian and Uruguayan samples of *L. colocola* and all *L. tigrinus* (Figure 2). Brazilian and Uruguayan populations formed the most recent monophyletic cluster based on DSA, and the second based on DSB, with estimated TMRCA dating from 266 to 287 kya, with a 95% confidence interval of 164 – 406 kya. However, the two data sets resulted in different views of the phylogenetic relationships between this group and those found in the western portion of South America. DSA indicated reciprocal monophyly between western and eastern populations, while DSB rendered the western region paraphyletic in relation to eastern populations. Cossios *et al.* (2009) also observed this same paraphyletic pattern, and considering that DSB includes a broader geographic representation of *L. colocola* genetic variation, we consider that this phylogenetic resolution is more plausible at the present time.

Within the Brazilian/Uruguayan cluster, two major clades could be recognized, each of them with an internal subdivision into two subgroups, leading to the identification of four different clusters. Clusters 1 and 3 (Figure 2) contain only haplotypes sampled in *L. colocola,* with the former including specimens from central and northeastern Brazil, in addition to one sample from Catamarca/Argentina and one sample of *L. tigrinus* from northeastern Brazil. Cluster 3, on the other hand, included all the haplotypes sampled in *L. colocola* individuals from southern Brazil and Uruguay. Haplotypes found in *L. tigrinus* were subdivided into Clusters 2 and 4, with the majority of *L. tigrinus* from northeastern Brazil being associated to Cluster 2 and *L. tigrinus* from central Brazil to Cluster 4 (Figure 2).

The estimated TMRCAs indicated that the divergence of haplotypes from central and northeastern Brazilian *L. colocola* occurred before the southern Brazilian and Uruguayan diversification, with a mean value of 92 kya for DSA and 104 kya for DSB in the first population, and 66 kya by both data sets for the second population. Divergence of haplotypes found in *L. tigrinus* seems to have occurred approximately at the same time, or shortly before it, with estimated dates ranging from 118 to 70 kya.

Haplotypes from western South America comprised the basal groups and were subdivided into several clusters: 5 and 6 for DSA and 5 – 9 for DSB. A detailed evaluation of DSB indicated that the most basal groups (7, 8 and 9) were restricted to areas in the southern portion of western South America (south of latitude 19º S), including Argentina, Chile and southern Bolivia. Cluster 6 was comprised uniquely by haplotypes sampled in the northern portion of western South America (north of latitude 19º S). Cluster 5 grouped the haplotypes most related to the eastern samples, and presented a mixed composition with individuals sampled in the northern and southern portions of western South America. In particular, this cluster contained samples from latitudes 17 – 25° S, in a partially intermediate geographic position between the northern and southern groups.

### Haplotype relationships

**Figure 3:**
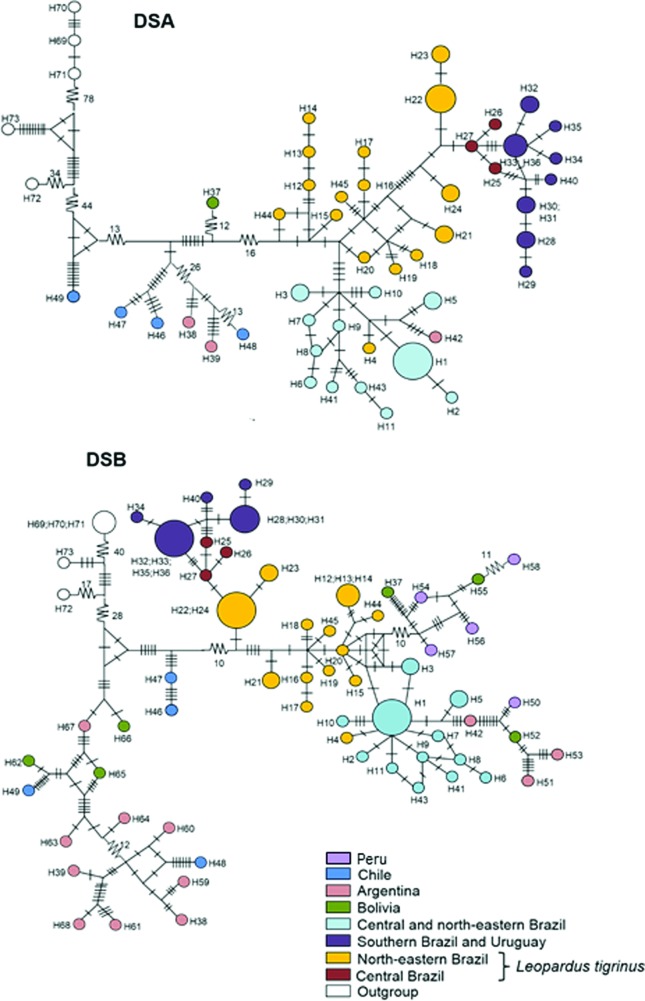
Haplotype networks for mitochondrial DNA segments sampled in *Leopardus colocola* and *L. tigrinus.* The data consist of concatenated sequences from the mitochondrial *ATP8*, *Cytb,* Control Region and *ND5* segments. Haplotypes are represented by circles whose size is proportional to their frequency. Lines on branches indicate the number of mutational steps between haplotypes; differences larger than 10 mutational steps are indicated by numbers. The top panel depicts the results obtained with data set A (DSA), encompassing longer sequences; the bottom panel depicts results based on data set B (DSB), which incorporates additional, shorter sequences reported by Cossíos *et al.* (2009).

The haplotype networks indicated a similar geographic structure to that observed in the phylogenetic analyses for eastern South America (SA) populations, with central and northeastern *L. colocola* segregated from southern Brazil and Uruguay, and also from *L. tigrinus* (Figure 3A,B). Interestingly, haplotypes found in *L. tigrinus* samples showed a more basal position in both networks in relation to those found in Brazilian and Uruguayan *L. colocola*. In the DSB network (calculated with complete deletion of sites with missing information; Figure 3B), the sequences contained in the groups of central and northeastern Brazil (including *L. colocola* and *L. tigrinus* specimens) had a star-shaped pattern, suggesting a possible demographic expansion, which could be connected to the inferred haplotype diversification.

As also evidenced in the phylogenetic analyses, haplotypes from western SA appeared as the most basal lineages in the phylogeographic structure of *L. colocola*. For DSA, one haplotype originating from northern Chile (H49) was the most basal, while for DSB, the basal position was occupied mainly by haplotypes from southern portions of the Andes (below latitude 19° S, including Bolivia, Argentina and Chile).

### Genetic structure

Genetic structure and gene flow analyses were only performed for DSB, due to its broader coverage of the *L. colocola* geographic distribution. The results from BAPS analyses performed with DSB were initially unstable and identified 4-5 genetic groups. In all of the five runs performed, two groups were stable and in agreement with the phylogenetic and network analyses, including mostly haplotypes sampled in eastern SA. Haplotypes from western SA were subdivided into 2 or 3 different groups, with an unstable allocation of individuals among different runs.

**Figure 4:**
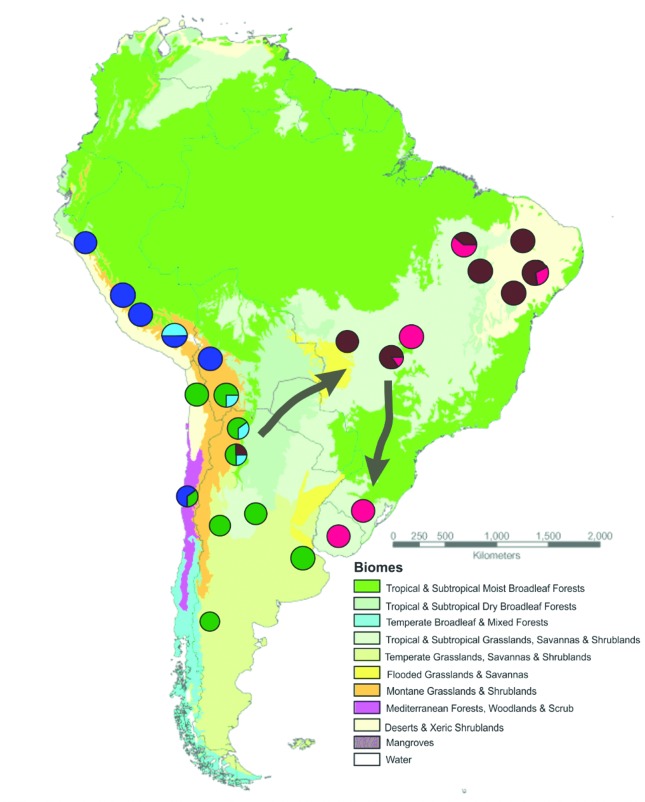
Map of South America showing the major genetic groups identified throughout the analyzed *L. colocola* distribution. The circles represent each sampling location, and the colors indicate the relative presence of haplotypes associated with each of the genetic groups identified with the BAPS analysis: Group 1 – central and north-eastern Brazilian *L. colocola* + *L. tigrinus* (brown); Group 2 – southern Brazilian and Uruguayan *L. colocola* + *L. tigrinus* (pink); Group 3 – south-western *L. colocola* (green); Group 4 – north-western *L. colocola* (dark blue); and Group 5 – central-western *L. colocola* (light blue). The arrows indicate the predominant direction of migration between populations based on analyses performed with the software LAMARC. Neotropical biomes are defined according to Olson *et al.* (2001).

Considering that these samples comprised mainly the shorter sequences reported by Cossíos *et al.* (2009), we decided to perform the same analyses with only the segments available for all samples (575 bp), in order to remove some possible noise induced by a large amount of missing data. This strategy led to stable results, with five groups identified with the same combination of haplotypes across all five runs: Group 1 (CNB), including all haplotypes from Clusters 1 and 2 (see Figure 2B) formed by central and northeastern Brazilian samples; Group 2 (SBU), comprising Clusters 3 and 4 with southern Brazilian and Uruguayan samples; Group 3 (SW), with haplotypes sampled in southwestern SA below latitude 19° S, and corresponding to Clusters 7 and 8 in Figure 2B; Group 4 (NW) with haplotypes from northwestern SA (above latitude 19° S), corresponding to Cluster 6, in addition to the two haplotypes from central Chile (H46 and H47), which formed Cluster 9 in the phylogenetic analyses; and Group 5 (CW), includind a mixture of haplotypes from the northern and southern parts of western SA (Cluster 5 in Figure 2B) being named here as the central-western population (Figure 4).

Levels of genetic differentiation among these five groups were evaluated through Φ_*ST*_ indices. All resulting values were statistically significant and considered high, indicating a strong genetic differentiation between these clusters (Table 2). The highest Φ_*ST*_ values were obtained in comparisons between the two eastern populations (CNB and SBU) and two of the three western populations (NW and CW), with SW being the western group most closely related to the eastern groups. The lowest Φ_*ST*_ values were found, in general, in comparisons between different western groups, except for NW *vs.* CW. This was quite intriguing, given the geographic proximity between these groups, but the results obtained for the Mantel test analysis also reflected this pattern. In spite of significant correlations among genetic and geographic distance (full data set: *r* = 0.337, *P* = 0.000; eastern group: *r* = 0.276, *P* = 0.000; western group: *r* = 0.281, *P* = 0.000), the correlation was relatively low mainly due to the occurrence of haplotypes with higher genetic distance but very close geographic origin (see Supplemental Figure S1).

**Table 2:** Pairwise Φ_*ST*_ values between defined geographical populations for *L. colocola.*

	CNB	SB U	NW	SW	CW
CNB	-				
SB U	0.550	-			
NW	0.663	0.813	-		
SW	0.587	0.578	0.397	-	
CW	0.620	0.845	0.614	0.423	-

CNB – Central and north-eastern Brazil; SB U – Southern Brazil and Uruguay; NW – north-western South America; SW – South-western South America; CW – Central-western South America.

* p < 0.0001 for all comparisons.

### Gene flow

Historical patterns of migration among populations were estimated for a scheme with only four populations. This included the groups identified with the BAPS analysis, with the exclusion of the CW group due to its small sample size. The haplotypes assigned to this group were allocated to the two other subpopulations of western SA according to their geographic origin in relation to latitude 19° S. Effective migration was estimated to be less than one migrant per generation, indicating limited dispersal and gene flow (Table 3). Two predominant directions of migration were observed using DSB, one from western to eastern regions of South America, and another from central and northeastern Brazil to southern Brazil and Uruguay (Table 3 and Figure 4). Estimates of migrant numbers yieded the highest values between NW and SW, CNB and SBU, and CNB and SW (Table 3).

**Table 3:** Estimation of migration rates between geographical populations of *L. colocola* based on mitochondrial DNA from data set B (DSB). Migration rate is scaled by mutation rate per site per generation. Nm is the estimated number of migrants entering a population per generation, and is obtained by multiplication of migration rates *vs.* theta for the receiving population. The left columns show the highest migration rates estimated for each pair of population, showing the predominant migration directions from western to eastern regions of South America, and from central and northeastern Brazil to southern Brazil and Uruguay.

Source population	Receiving population	Migration (95% confidence interval)	Nm	Source population	Receiving population	Migration (95% confidence interval)	Nm
NW	CNB	5.499 (0.010 – 24.701)	0.154	CNB	NW	5.149 (0.010 – 24.289)	0.293
SW	CNB	7.552 (0.010 – 30.288)	0.211	CNB	SW	1.991 (0.010 – 9.288)	0.234
NW	SBU	32.725 (0.010 – 158.245)	0.087	SBU	NW	4.171 (0.010 – 21.359)	0.237
SW	SBU	42.116 (0.010 – 210.927)	0.112	SBU	SW	2.057 (0.010 – 9.836)	0.242
SW	NW	10.907 (0.010 – 44.687)	0.619	NW	SW	6.119 (0.010 – 18.975)	0.721
CNB	SBU	82.371 (0.010 – 312.711)	0.219	SBU	CNB	15.002 (0.010 – 45.779)	0.420

NW – North-western South America

SW – South-western South America;

CNB – Central and North-eastern Brazil

SBU – Southern Brazil and Uruguay

### Demographic history

Mismatch distribution analyses and neutrality tests were used to test the hypothesis of a recent population expansion in *L. colocola*. For this, we used only DSB. The mismatch distribution for the entire data set resulted in a bimodal graph, which would be consistent with a heterogeneous genetic composition of the data set. The analyses conducted with western and eastern regions independently revealed a unimodal pattern (although slighly irregular) only for the former, with a mode around 30 differences suggesting a rapid and relatively old population expansion (see Figure S2). On the other hand, Fu’s *F*_S_ neutrality test resulted in negative and significant values for both populations analyzed independently and also for the entire data set (entire data set: -24.11; eastern: -15.91; western: -11.17; p < 0.05), indicating possible events of demographic expansion in all the assessed data sets.

**Figure 5:**
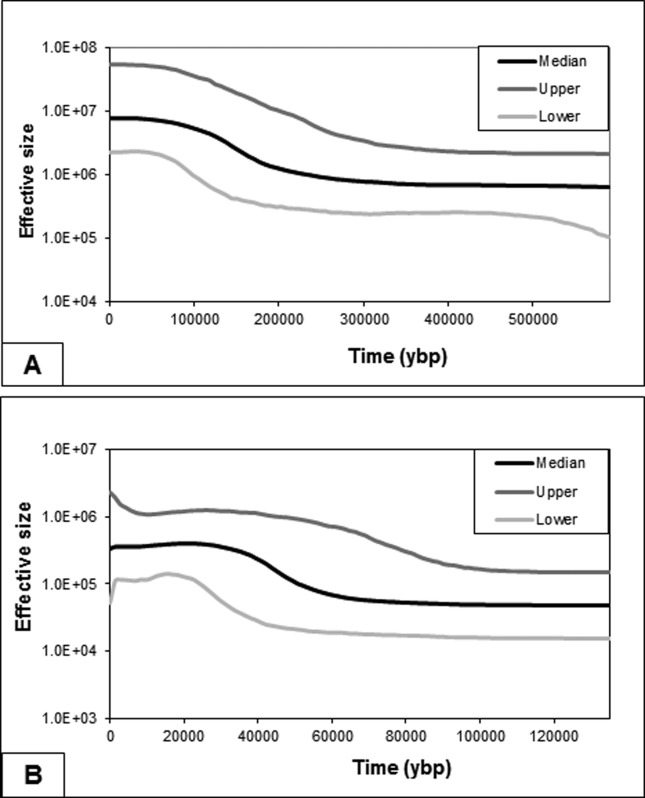
Bayesian skyline plot showing the effective population size fluctuation of *L. colocola* through time. (A) Analysis performed with all haplotypes from western South America and (B) Analysis performed with all haplotypes from eastern South America.

The Bayesian Skyline plot showed a constant population size until about 300 kya, with a strong signal of population expansion in recent times. For western SA, we observed a strong signal of population expansion starting around 200 kya (Figure 5A). On the other hand, for the eastern SA group, a more recent signal of demographic expansion was detected around 60 – 50 kya (Figure 5B).

## Discussion

### Phylogenetic relationships and genetic structure

Mitochondrial analyses revealed a strong genetic structure across the *L. colocola* distribution, in agreement with previous studies of this species (Johnson *et al.*, 1999; Napolitano *et al.*, 2008; Cossíos *et al.*, 2009). Several monophyletic clusters were identified in the phylogenetic analyses, in strong concordance with the groups found by Cossíos *et al.* (2009) for the central Andes. According to the BAPS analyses, these clusters could be joined into five main genetic groups distributed in west-east and north-south directions. However, these lineages were not completely allopatric, given the co-occurrence of unrelated haplotypes at some localities, mainly in central South America (see Figure 4), as was also reported by Cossíos *et al.* (2009) and Napolitano *et al.* (2008). This pattern suggests that this particular region presents a more complex history that favored events of longstanding isolation with posterior contact between different populations at different times during the evolution of this species.

Historical connections between the western and eastern portions of the pampas cat distribution seem to have mainly occurred via southern populations of the west. Migration analyses indicated that the highest effective migration occurred from western to eastern populations, especially to central and north-eastern Brazil, probably taking the diagonal dry corridor formed by the interconnection of three tropical/subtropical open biomes: Caatinga (a seasonally dry tropical forest in northeastern Brazil), Cerrado (central Brazilian savanna), and Chaco (in northeastern Argentina, western Paraguay and south-eastern Bolivia) (Werneck, 2011). Several studies document the importance of this biome connection to the migration and gene flow of open-habitat vertebrates (Werneck *et al.*, 2012 and references therein), and our data suggest that this region may have been important for the establishment of the historical relationships between western and eastern pampas cat populations. A high migration rate was also estimated for the eastern portion of South America, from central/northeastern Brazil to southeastern Brazil/Uruguay. In the past, this historical migration could be favored during glacial periods associated with the reduction of forested environments (Behling, 1998, 2002) that subsequently have likely isolated these two populations.

In spite of the migration results, recent gene flow between the five lineages identified here seems to be relatively low, taking into account their high genetic differentiation, especially between eastern and western regions of South America. In fact, although haplotype co-occurrence was identified at some localities, the five lineages identified here were almost completely segregated geographically.

Among the three major groups identified in western South America, the northwestern one was mainly associated to the Montane Grasslands of Peru and northern Bolivia, while the southwestern one was documented in a variety of biomes, including the temperate grasslands (Patagonian Steppe), Mediterranean Forests and Montane grasslands of Argentina, southern Bolivia and Chile. The boundary between these two populations apparently lies around parallel 18-19° S as was also documented by Cossíos *et al.* (2009). The third group (central-western) was identified around 17° – 25° S, at an intermediate position between the former two populations, in a transitional zone between highland steppes to dry forested lowlands (Garcia-Perea, 1994).

The latitudes comprising the central-western population and the limit between the occurrence of the north and southwestern ones, approximately correspond to a specific dry subtropical region of the Andes, known as the “dry diagonal” (Ammann *et al.*, 2001; Kull *et al.*, 2002). According to those authors, this area is characterized by extremely dry conditions and constitutes a transitional zone between two circulation systems found in South America, which have specific impacts on the climate of this area, leading to a northern region with higher precipitation and a southern region with low precipitation. These different local environmental conditions in the north and south, an also inside the dry diagonal, may have favored the evolution of differently adapted forms. In fact, this region has already been suggested to have played an important role in determining the genetic structure of other Andean species, such as the vicuña (*Vicugna vicugna*) (Marín *et al.*, 2007). Interestingly, genetic differentiation found between the north and southwestern populations was lower than those found between these same populations and the central-western group that seems to be geographically closer. This pattern might indicate that some historical gene flow was present between the northern and southern populations along the Andes, with longer periods of isolation relative to the central-western population.

In eastern regions of pampas cat occurrence, the two identified populations were also subdivided according to different biomes. Northern populations are restricted to two Brazilian biomes, Pantanal (humid grasslands connected to the Chaco biome [although there is no evidence of *L. colocolo* in the Chaco – Cuellar *et al.*, 2006, Lucherini *et al.*, 2016]) and Cerrado, while southern populations are restricted to the Pampas steppes of southern Brazil, Uruguay e northeastern Argentina. Although higher values of genetic differentiation were generally found between eastern and western populations, the genetic differentiation found between these eastern lineages was also quite high. In fact, no haplotype found in *L. colocola* specimens was shared or found in sympatry between these two populations (exceptions included only the haplotypes found in *L. tigrinus* individuals who carried some haplotypes from the southern group).

Probably the gene flow between these two populations was interrupted by the return of forested environments in Brazil around 17 kya in the Late Pleistocene (Ledru *et al.*, 1996). In addition, the southeastern population restricted to the Pampas biome seems to be the most isolated unit under present conditions. No geographic population shared any haplotype with this unit, and despite its geographic proximity to the southwestern group, a high genetic differentiation was found between these populations. Although the absence of haplotype sharing with other regions could be only due to a sampling artifact, current geographic conditions contribute to the interpretation of an isolated population, including several possible geographical barriers, such as the Atlantic Forest on the north, the Uruguay, Paraguay and Paraná rivers on the west and La Plata River on the south.

Finally, our data corroborate those reported by Johnson *et al.* (1999), by indicating that the Andean region may not present a strong barrier to this species, since haplotypes from the same lineage/group were found on both sides of the Cordillera. This is not necessarily surprising, since the Andean Cordillera seems also not to be an effective barrier to gene flow even for some small rodents (e.g., Smith *et al.*, 2001; Palma *et al.*, 2005; Rodriguez-Serrano *et al.*, 2006; Cañón *et al.*, 2010). On the other hand, the Paraná River, a tributary of the Plata River Basin, seems to be a more effective barrier for *L. colocola*, as it is for other large mammals, such as the marsh deer *Blastocerus dichotomus* (Márquez *et al.*, 2006).

### Demographic history and hybridization/introgression with *L. tigrinus*

Two episodes of demographic expansion were detected by our analyses: one older in western South America (around 200 kya), and one more recent in eastern SA (60-50 kya). These episodes were supported by the skyline plots and neutrality tests, even though they were not as visible in the mismatch distribution analyses. This latter result could be attributed to an insufficient sampling, or a more complex demography history in addition to a simple event of population expansion. In fact, the haplotype networks depicted few cases consistent with an expansion pattern, indicating a more complex history of pampas cat populations. The estimated ages for these demographic events, in addition to the results gleaned from the networks, phylogenetic and migration rate analyses, support the inference that ancestral haplotypes from pampas cat occurred in western South America, especially in the southwestern portion of the continent. Nevertheless, additional evidence from the haplotype networks illustrate the complexity of pampas cat mtDNA history, with lineages/groups found in the north and central-west areas apparently derived from those found in central Brazil. This phylogeographic pattern suggests a demographic colonization from southwestern to central Brazil, possibly via the dry corridor, with a posterior colonization of the western regions in the northern part of the continent.

The estimated age for both events of population expansion were coincident with two independent Pleistocene colder periods (Ehlers and Gibbard, 2007). This Quaternary epoch seems to play an important role in the biogeographic pattern of several organisms, mainly due to the strong climatic fluctuations inferred for that time, with important consequences on environment conditions (Hewitt, 1996, 2001, 2004). In general, interglacial periods characterized by more humid and warmer conditions were associated with demographic expansion of forest-adapted species. However, the association of colder and drier conditions of the glacial periods with demographic expansion of open-habitat organism is not so clear, with different species presenting variable responses to these climatic oscilations (Turchetto-Zolet *et al.*, 2013).

In Brazil, the age of the most recent expansion inferred for *L. colocola* is coincident with a colder and drier period in the country, which was associated with a retraction of forested environments and expansion of subtropical grasslands (Ledru *et al.*, 1996, Behling, 1998, 2002). Additionally, some studies have also demonstrated a possible correlation between grassland expansions favored in glacial periods and geographic movements of open-habitat plants (Jakob *et al.*, 2009; Cosacov *et al.*, 2010) and animals (Wüster *et al.*, 2005; Marín *et al.*, 2007; Prado *et al.*, 2012). Therefore, we conclude that this open-habitat cat may have expanded its geographic distribution into previously unavailable areas, following the estabilishment of more open habitats.

The demographic history of *L. colocola* became even more complex when we examined the haplotypes found in *L. tigrinus* individuals. Interestingly, these haplotypes seem to be ancestral to those currently found in *L. colocola* from Brazil, Uruguay and north and central-western populations, thus constituting an important record of the evolutionary history of this species. This pattern, in addition to the absence of haplotype sharing between *L. tigrinus* and *L. colocola* individuals, is highly intriguing and suggests that the introgression process from the latter species into *L. tigrinus* is quite old. The recognition of an ancient process would explain the intermediate position of *L. tigrinus* haplotypes, indicating that introgression events occurred before the geographic expansion and differentiation of eastern populations. In fact, according to our estimated dates, the diversification of *L. colocola* haplotypes from eastern South America occurred at a similar time as the diversification of *L. tigrinus* haplotypes (mean values around 118 – 70 kya for *L. tigrinus* and 100 – 60 kya for *L. colocola*). Therefore, we postulate that hybridization/introgression events occurred in central Brazil soon after the colonization of eastern South America, thus being connected with the first demographic expansion detected in this study. The *L. colocola* haplotypes present in the individuals that arrived in eastern SA seem to remain only (likely bearing additional mutations) in the *L. tigrinus* population, and may have become extinct in the current eastern populations of *L. colocola*.

Whether hybridization between *L. tigrinus* and *L. colocola,* leading to complete mitochondrial introgression into the former species, occurred before, during, or after the divergence of *L. tigrinus* and *L. guttulus* is still uncertain. Available estimates for divergence of these species are 103 kya – 1.038 Mya (Trigo *et al.*, 2013). This estimation would indicate that *L. tigrinus* and *L. guttulus* diverged before the introgression from *L. colocola* into the former. However, this estimate was obtained based on only two introns of the Y chromosome containing few variable sites, leading to an extremely broad confidence interval. Considering the minimum value (103 kya), hybridization and introgession from *L. colocola* could have occurred at an early stage of the divergence between the two tigrina species, and thus could have played a role in this divergence process.

### Taxonomic considerations

Three distinct species have been proposed for the pampas cat by Garcia-Perea (1994): *Leopardus colocolo*, *Leopardus pajeros* and *L. braccatus*. This proposed classification was followed by Wozencraft (2005) and extended by Nascimento (2010, Doctoral thesis, Universidade de São Paulo, São Paulo). Previous molecular studies (Johnson *et al.*, 1999; Napolitano *et al.*, 2008; Cossios *et al.*, 2009) did observe strong population structure but did not find support for this species-level categorization, given the recent estimated age of the inferred phylogroups. These earlier molecular studies were based on limited geographic coverage, which in some cases precluded a more comprehensive comparison with the morphological proposals. Given the broader sampling we achieved in the present study, we attempt here an extended comparison with specific and subspecific categories proposed for the pampas cat group.

*L. colocolo* recognized by Garcia-Perea (1994) was subdivided into two subspecies: *L. c. wolffsohni* from the highland steppes of northern Chile and *L. c. colocolo* from forests of central Chile. According to our results, samples collected in northern Chile harbored a haplotype that is clearly connected to populations found in the southwestern region on the eastern slope of the Andes, in a group that is recognized as *L. pajeros* by Garcia-Perea (1994) *.* In addition, Cossíos *et al.* (2009) demonstrated that sequences obtained by Napolitano *et al.* (2008) for northern Chile form a clear monophyletic group with haplotypes representing the central-western population identified by our data. These findings indicate that the population in northern Chile, in spite of the Andean Cordillera, is closely related to pampas cat from the eastern Andes, thus not supporting its recognizition as a distinct species.

On the other hand, samples evaluated here from central Chile, corresponding to *L. c. colocolo*, present more complex relationships. While one sample was clearly associated to the southwestern group, in a pattern similar to that obtained from northern Chile across all analyses, two other samples were more closely assigned to southern or northwestern populations, depending on the analysis performed. These results indicate a more unstable position of this population, requiring a larger sample to define its phylogenetic position more precisely.

For *L. pajeros*, seven subspecies were recognized by Garcia-Perea (1994), although the author herself recognized that only three groups (northern, central and southern) could be found along this taxon’s geographic distribution, taking into account habitat and morphological similarities. The northern portion of the *L. pajeros* distribution was represented by three subspecies: *L. p. thomasi* from Ecuador, *L. p. garleppi* from Peru and *L. p. steinbachi* from Bolivia. Unfortunately, we did not have access to samples representing *L. p. thomasi* and *L. p. steinbachi,* but the geographic distribution of *L. p. garleppi* is in great concordance with the northwestern group identified here, with only a proposed extension of this distribution to regions of northwestern Bolivia. According to Garcia-Perea (1994), *L. p. thomasi* and *L. p. garleppi* have similar pelage patterns and cranial morphology and occurr in the same habitats, and could thus be assigned to the same geographic population. *L. p. steinbachi* presents only slight morphological differences from *L. p. garlepii*, and given its geographic proximity to the limit of the northwestern group found in this study, this taxon might also be a synonym of *garleppi*.

The central and southern distribution of *L. pajeros* includes the subspecies *L. p. budini* and *L. p. crespoi* from montaneous areas of northwestern Argentina, *L. p. pajeros* from central Argentina with a possible boundary in the Pampa Province of Argentina, around latitude 38°S, and *L. p. crucinus* from southern Chile and Argentina. *L. p. crespoi* is only known from the type specimen and Garcia-Perea (1994) considered that this may be a synonym of *L. p. budini* due to morphological and habitat similarities. The geographic distribution of these two subspecies approximates the central-western population identified in our study, in a transitional area between highland steppes and lowland dry forests. The geographic distribution of *L. p. pajeros* strongly corresponds to the southern group found in our analyses, with only an extension of its distribution over that which would be recognized as *L. p. crucinus,* approximately around latitude 41° S. However, additional sampling from areas south of this area would be important to evaluate the existence of an additional subspecies in southernmost Argentina and Chile.

The eastern populations of pampas cat were subdivided by Garcia-Perea (1994) into two subspecies of *L. braccatus*: *L. b. braccatus* for Central Brazil and *L. b. munoai* for southern Brazil, Uruguay and northeastern Argentina. According to that author, morphological differences in skulls are not clear for these subspecies, but there are relevant distinctions in coat pattern and body size. In agreement with this view, our results indicate a strong genetic differentiation between these populations, indicating that they consist of two separate evolutionary units.

Overall, our study allowed the identification of at least five genetic groups in *L. colocola*, which were largely consistent with currently recognized subspecies (Kitchener *et al.*, 2017). This organization is also mostly in agreement with the results obtained by Nascimento (2010, Doctoral thesis, Universidade de São Paulo, São Paulo), although that author considered all these populations to represent distinct species. Based on our data, the pampas cat can so far be viewed conservatively as a complex species with marked population structure across its range. The evolutionary history of this cat seems to have been defined by vicariant isolation of lineages during lengthy periods, followed by population expansion into new habitats or into previously inhabited areas, leading to new secondary contact zones and the complex genetic patterns observed here. Additional analyses, integrating mitochondrial and nuclear data, as well as detailed morphological and ecological information, will be required to further assess the hypothesis that these regional pampas cat populations have achieved species-level distinctiveness. Regardless of the ultimate resolution of this taxonomic discussion, for conservation purposes these regional populations should be considered distinct Evolutionarily Significant Units (ESUs), and thus be the focus of separate assessment and management actions.

## Supplementary material

The following online material is available for this article:

Table S1 - Samples and sequences analyzed in the present study.

Figure S1 - Correlation between genetic and geographic distance for *L. colocola* and *L. tigrinus* haplotypes.

Figure S2 - Analysis of the pairwise nucleotide differences distribution (Mismatch Distribution) of mtDNA haplotypes assessed in this study.

## References

[B1] Akaike H (1974). A new look at the statistical identification model. IEEE T Automat Contr.

[B2] Ammann C, Jenny B, Kammer K, Messerli B (2001). Late Quaternary Glacier response to humidity changes in the arid Andes of Chile (18-29^o^S). Palaeogeogr Palaeoclimatol Palaeoecol.

[B3] Bandelt HJ, Forster P, Röhl A (1999). Median-joining networks for inferring intraspecific phylogenies. Mol Biol Evol.

[B4] Behling H (1998). Late Quaternary vegetational and climatic changes in Brazil. Rev Palaeobot Palynol.

[B5] Behling H (2002). South and Southeast Brazilian grasslands during Late Quaternary times: A synthesis. Palaeogeogr Palaeoclimatol Palaeoecol.

[B6] Cañón C, D’Elia G, Pardinas UFJ, Lessa EP (2010). Phylogeography of *Loxodontomys micropus* with comments on the alpha taxonomy of *Loxodontomys* (Cricetidae: Sigmodontinae). J Mammal.

[B7] Corander J, Tang J (2007). Bayesian analysis of population structure based on linked molecular information. Math Biosci.

[B8] Corander J, Marttinen P, Sirén J, Tang J (2008). Enhanced Bayesian modeling in BAPS software for learning genetic structures of populations. BMC Bioinformatics.

[B9] Cosacov A, Sersic AN, Sosa V, Johnson LA, Cocucci AA (2010). Multiple periglacial refugia in the Patagonian steppe and post-glacial colonization of the Andes: the phylogeography of *Calceolaria polyrhiza*. J Biogeogr.

[B10] Cossíos D, Lucherini M, Ruiz-García M, Angers B (2009). Influence of ancient glacial periods on the Andean fauna: The case of the pampas cat (*Leopardus colocolo*). BMC Evol Biol.

[B11] Cuellar E, Maffei L, Arispe R, Noss A (2006). Geoffroy’s cats at the northern limit of their range: activity patterns and density estimates from camera trapping in Bolivian dry forests. Stud Neotrop Fauna Environ.

[B12] Drummond A, Rambaut A (2007). BEAST: Bayesian evolutionary analysis by sampling trees. BMC Evol Biol.

[B13] Edgar RC (2004). MUSCLE: multiple sequence alignment with high accuracy and high throughput. Nucleic Acids Res.

[B14] Ehlers J, Gibbard PL (2007). The extent and chronology of Cenozoic Global Glaciation. Quatern Int.

[B15] Eisenberg JF, Redford KH (1999). Mammals of the Neotropics. The Central Tropics: Ecuador, Peru, Bolivia, Brazil.

[B16] Eizirik E, Bonatto SL, Johnson WE, Crawshaw PG, Vié JC, Brousset DM, O’Brien SJ, Salzano FM (1998). Phylogeographic patterns and evolution of the mitochondrial DNA control region in two Neotropical cats (Mammalia, Felidae). J Mol Evol.

[B17] Eizirik E, Patterson BD, Costa LP (2012). A molecular view on the evolutionary history and biogeography of Neotropical carnivores (Mammalia, Carnivora). Bones, Clones and Biomes: The History and Geography of Recent Neotropical Mammals.

[B18] Excoffier L, Lischer HEL (2010). Arlequin suite ver 3.5: A new series of programs to perform population genetics analyses under Linux and Windows. Mol Ecol Resour.

[B19] Excoffier L, Smouse P, Quattro J (1992). Analysis of Molecular Variance inferred from metric distances among DNA haplotypes: application to human mitochondrial DNA restriction data. Genetics.

[B20] Fu YX (1997). Statistical test of neutrality of mutations against population growth, hitchhiking and background selection. Genetics.

[B21] Garcia-Perea R (1994). The Pampas Cat Group (Genus *Lynchailurus* Severtzov, 1858) (Carnivora: Felidae), a systematic and biogeographic review. Am Mus Novit.

[B22] Godoi MN, Teribele R, Bianchi RC, Olifiers N, Concone HVB, Xavier NLF (2010). New records of pampas cat (Leopardus colocolo, Molina 1782) for Mato Grosso do Sul State, Brazil. Cat News.

[B23] Guindon S, Gascuel O (2003). A simple, fast, and accurate algorithm to estimate large phylogenies by maximum likelihood. Syst Biol.

[B24] Guindon S, Dufayard JF, Lefort V, Anisimova M, Hordijk W, Gascuel O (2010). New algorithms and methods to estimate maximum-likelihood phylogenies: Assessing the performance of PhyML 3.0. Syst Biol.

[B25] Hewitt GM (1996). Some genetic consequences of ice ages, and their role in divergence and speciation. Biol J Linnean Soc.

[B26] Hewitt GM (2001). Speciation, hybrid zones and phylogeography - or seeing genes in space and time. Mol Ecol.

[B27] Hewitt GM (2004). Genetic consequences of climatic oscillations in the Quaternary. Philos Trans R Soc Lond B Biol Sci.

[B28] Irwin DM, Kocher TD, Wilson AC (1991). Evolution of the cytochrome b gene of mammals. J Mol Evol.

[B29] Jakob SS, Martinez-Meyer E, Blattner FR (2009). Phylogeographic analyses and paleodistribution modeling indicate Pleistocene in situ survival of *Hordeum* species (Poaceae) in southern patagonia without genetic or spatial restriction. Mol Biol Evol.

[B30] Johnson WE, Culver M, Iriarte JA, Eizirik E, Seymour KL, O’Brien SJ (1998). Tracking the evolution of the elusive Andean Mountain Cat (*Oreailurus jacobita*) from mitochondrial DNA. J Hered.

[B31] Johnson WE, Eizirik E, Pecon-Slattery J, Murphy WJ, Antunes A, Teeling E, O’Brien SJ (2006). The late Miocene radiation of modern Felidae: A genetic assessment. Science.

[B32] Johnson WE, Pecon-Slattery J, Eizirik E, Kim J, Menotti-Raymond M, Bonacic C, Cambre R, Crawshaw P, Nunes A, Seuánez HN (1999). Disparate phylogeography patterns of molecular genetic variation in four closely related South American small cat species. Mol Ecol.

[B33] Kim JH, Eizirik E, O’Brien SJ, Johnson WE (2001). Structure and patterns of sequence variation in the mitochondrial DNA control region of the great cats. Mitochondrion.

[B34] Kitchener AC, Breitenmoser-Würsten C, Eizirik E, Gentry A, Werdelin L, Wilting A, Yamaguchi N, Abramov AV, Christiansen P, Driscoll C (2017). A revised taxonomy of the Felidae. The final report of the Cat Classification Task Force of the IUCN/SSC Cat Specialist Group. Cat News Special.

[B35] Koepfli KP, Wayne RK (1998). Phylogenetic relationships of otters (Carnivora: Mustelidae) based on mitochondrial cytochrome b sequences. J Zool (Lond).

[B36] Kuhner MK (2006). LAMARC 2.0: maximum likelihood and Bayesian estimation of population parameters. Bioinform Appl Note.

[B37] Kull C, Grosjean M, Veit H (2002). Modeling modern and late Pleistocene glacio-climatological conditions in the north Chilean Andes (29-30^o^S). Clim Change.

[B38] Ledru M, Braga PIS, Soubiès F, Fournier M, Martin L, Suguio K, Turcq B (1996). The last 50,000 years in the Neotropics (Southern Brazil): Evolution of vegetation and climate. Palaeogeogr Palaeoclimatol Palaeoecol.

[B39] Li G, Davis BW, Eizirik E, Murphy WJ (2016). Phylogenomic evidence for ancient hybridization in the genomes of living cats (Felidae). Genome Res.

[B40] Librado P, Rozas J (2009). DnaSP v5: A software for comprehensive analysis of DNA polymorphism data. Bioinformatics.

[B41] Lucherini M, Eizirik E, de Oliveira T, Pereira J, Williams RSR (2016). Leopardus colocolo. The IUCN Red List of Threatened Species.

[B42] Mantel N (1967). The detection of disease clustering and a generalized regression approach. Cancer Res.

[B43] Marín JC, Casey CS, Kadwell M, Yaya K, Hoces D, Olazabal J, Rosadio R, Rodriguez J, Spotorno A, Bruford MW (2007). Mitochondrial phylogeography and demographic history of the Vicuña: implications for conservation. Heredity.

[B44] Márquez A, Maldonado JE, González S, Beccaceci MD, Garcia JE, Duarte JMB (2006). Phylogeography and Pleistocene demographic history of the endangered marsh deer (*Blastocerus dichotomus*) from the Rio de la Plata Basin. Conserv Genet.

[B45] Miller MP (2005). ALLELES IN SPACE: Computer software for the joint analysis o interindividual spatial and genetic information. J Hered.

[B46] Napolitano C, Bennett M, Johnson WE, O’Brien SJ, Marquet PA, Barría I, Poulin E, Iriarte A (2008). Ecological and biogeographical inferences on two sympatric and enigmatic Andean cat species using genetic identification of faecal samples. Mol Ecol.

[B47] Nowell K, Jackson P (1996). Wilds Cats: Status Survey and Conservation Action Plan.

[B48] Olson DM, Dinerstein E, Wikramanayake ED, Burgess ND, Powell GVN, Underwood EC, D’Amico JA, Itoua I, Strand HE, Morrison JC (2001). Terrestrial ecoregions of the world: a new map of life on Earth. Bioscience.

[B49] Palma RE, Marquet PA, Boric-Bargetto D (2005). Inter-and intraspecific phylogeography of small mammals in the Atacama Desert and adjacent areas of northern Chile. J Biogeogr.

[B50] Pereira J, Varela D, Fracassi N (2002). Pampas cat in Argentina: Is it absent from the pampas?. Cat News.

[B51] Posada D (2008). jModelTest: Phylogenetic Model Averaging. Mol Biol Evol.

[B52] Prado CPA, Haddad CFB, Zamudio KR (2012). Cryptic lineages and Pleistocene population expansion in a Brazilian Cerrado frog. Mol Ecol.

[B53] Queirolo D, Almeida LB, Beisiegel B, Oliveira TG (2013). Avaliação do risco de extinção do gato-palheiro *Leopardus colocolo* (Molina, 1782) no Brasil. Biodiv Bras.

[B54] Ramirez-Soriano A, Ramos-Onsins SE, Rozas J, Calafell F, Navarro A (2008). Statistical power analysis of neutrality tests under demographic expansions, contractions and bottlenecks with recombination. Genetics.

[B55] Rodriguez-Serrano E, Cancino RA, Palma RE (2006). Molecular phylogeography of *Abrothrix olivaceus* (Rodentia: Sigmodontinae) in Chile. J Mammal.

[B56] Rogers AR, Harpending HC (1992). Population growth makes waves in the distribution of pairwise genetic differences. Mol Biol Evol.

[B57] Ronquist F, Huelsenbeck JP (2003). MrBayes 3: Bayesian phylogenetic inference under mixed models. Bioinformatics.

[B58] Ruiz-Garcia M, Payán CE, Hernández-Camacho JI (2003). Possible records of *Lynchailurus* in south-western Colombia. Cat News.

[B59] Saiki RK, Scharf S, Faloona F, Mullis KB, Horn GT, Erlich HA, Arnheim N (1985). Enzymatic amplification of beta-globin genomic sequences and restriction site analysis for diagnosis of sickle cell anemia. Science.

[B60] Sambrook J, Fritsch EF, Maniatis T (1989). Molecular Cloning.

[B61] Silveira L (1995). Notes on the distribution and natural history of the pampas cat, *Felis colocolo*, in Brazil. Mammalia.

[B62] Smith MF, Kelt DA, Patton JL (2001). Testing models of diversification in mice in the Abrothrix olivaceus/xanthorhinus complex in Chile and Argentina. Mol Ecol.

[B63] Tamura K, Peterson D, Peterson N, Stecher G, Nei M, Kumar S (2011). MEGA5: Molecular Evolutionary Genetics Analysis using maximum likelihood, evolutionary distance, and maximum parsimony methods. Mol Biol Evol.

[B64] Tchaicka L, Eizirik E, Oliveira TG, Cândido JF, Freitas TRO (2007). Phylogeography and population history of the crab-eating fox (*Cerdocyon thous*). Mol Ecol.

[B65] Trigo TC, Freitas TRO, Kunzler G, Cardoso L, Silva JCR, Johnson WE, O’Brien SJ, Bonatto SL, Eizirik E (2008). Inter-species hybridization among Neotropical cats of the genus *Leopardus*, and evidence for an introgressive hybrid zone between *L. geoffroyi* and *L. tigrinus* in southern Brazil. Mol Ecol.

[B66] Trigo TC, Schneider A, de Oliveira TG, Lehugeur LM, Silveira L, Freitas TR, Eizirik E (2013). Molecular data reveal complex hybridization and a cryptic species of neotropical wild cat. Curr Biol.

[B67] Turchetto-Zolet AC, Pinheiro F, Salgueiro F, Palma-Silva C (2013). Phylogeographical patterns shed light on evolutionary process in South America. Mol Ecol.

[B68] Villalba L, Delgado E (2005). Pampas cat photographed in High Southwest Bolivia. Cat News.

[B69] Werneck FP (2011). The diversification of eastern South American open vegetation biomes: historical biogeography and perspectives. Quat Sci Rev.

[B70] Werneck FP, Gamble T, Colli GR, Rodrigues MT, Sites JW (2012). Deep diversification and long-term persistence in the South American “Dry Diagonal”: integrating continent-wide phylogeography and distribution modeling of geckos. Evolution.

[B71] Wozencraft WC, Wilson DE, Reeder DM (2005). Carnivora. Mammal Species of the World: A Taxonomic and Geographic Reference.

[B72] Wüster W, Ferguson JE, Quijada-Mascarenas JA, Pook CE, Salomão MG, Thorpe RS (2005). Tracing an invasion: landbridges, refugia, and the phylogeography of the Neotropical rattlesnake (Serpentes: Viperidae: *Crotalus durissus*). Mol Ecol.

