# Supplementary material for: Phylogeographic analyses of the pampas cat (Leopardus colocola; Carnivora, Felidae) reveal a complex demographic history
Source: Genet Mol Biol. 2018;41(1 Suppl 1):273–87. doi: 10.1590/1678-4685-GMB-2017-0079 (PMC5913729; doi:10.1590/1678-4685-GMB-2017-0079)
Supplement: Supplementary file 1 [file 1415-4757-GMB-41-01-2017-0079-s001.pdf]

Supplementary Material to "Phylogeographic analyses of the pampas cat (*Leopardus colocola*; Carnivora, Felidae) reveal a complex demographic history"

Table S1 - Samples and sequences analyzed in the present study with the description of the haplotypes found for the four analyzed mitochondrial segments (*ATP8*, *Cytb*, *Control Region* and *ND5*).

Samples	Haplotype	Geographic origin	Institution/contact
bLco301 [A, N, C*, RC], bLco302 [A, N, C, RC], bLco311 [A, N, C, RC], bLco312 [A, N, C, RC], bLco317 [A, N, C, RC], bLco320 [A, N, C, RC]	H1	Goiás/ Central Brazil	Leandro Silveira, Anah Jácomo, Mariana Furtado, Cynthia Kashivakura
Lco30 [A, N, C, RC]	H2	Unknown origin	NCI-USA
bLco309 [A, N, C, RC], bLco310 [A, N, C, RC]	H3	Goiás/ Central Brazil	Leandro Silveira, Anah Jácomo, Mariana Furtado, Cynthia Kashivakura
bLti150 [A, N, C, RC]	H4	Ceará/Northeastern Brazil	Tadeu Gomes de Oliveira
bLco305 [A, N, C, RC], bLco314 [A, N, C, RC]	H5	Goiás/ Central Brazil	Leandro Silveira, Anah Jácomo, Mariana Furtado, Cynthia Kashivakura
bLco307 [A, N, C, RC]	H6	Goiás/ Central Brazil	Leandro Silveira, Anah Jácomo, Mariana Furtado, Cynthia Kashivakura
Lco13 [A, N, C, RC]	H7	Goiás/ Central Brazil	Zoológico de São Paulo (NCI-USA)
bLco306 [A, N, C, RC]	H8	Goiás/ Central Brazil	Leandro Silveira, Anah Jácomo, Mariana Furtado, Cynthia Kashivakura
bLco308 [A, N, C, RC]	H9	Goiás/ Central Brazil	Leandro Silveira, Anah Jácomo, Mariana Furtado, Cynthia Kashivakura
bLco09 [A, N, C, RC]	H10	Goiás/ Central Brazil	Maurício Barbanti
bLco24 [A, N, C, RC]	H11	Maranhão/ Northeastern Brazil	Carlos Benhur Kasper
bLti107 [A, N, C, RC]	H12	Piauí/ Northeastern Brazil	Criadouro de Morro Reuter
bLti304 [A, N, C, RC]	H13	Piauí/ Northeastern Brazil	Zoológico de Teresina
bLti148 [A, N, C, RC]	H14	Maranhão/ Northeastern Brazil	LBGM – PUCRS
bLti166 [A, N, C, RC]	H15	Pernambuco/ Northeastern Brazil	Tadeu Gomes de Oliveira

Samples	Haplotype	Geographic origin	Institution/contact
bLti129 [A, N, C, RC]	H16	Piauí/ Northeastern Brazil	M. Reis / IBAMA
bLti154 [A, N, C, RC]	H17	Maranhão/ Northeastern Brazil	Tadeu Gomes de Oliveira
bLti130 [A, N, C, RC]	H18	Piauí/ Northeastern Brazil	M. Reis / IBAMA
bLti218 [A, N, C, RC]	H19	Sergipe/ Northeastern Brazil	Zoológico de Aracajú
bLti153 [A, N, C, RC]	H20	Ceará/Northeastern Brazil	Tadeu Gomes de Oliveira
bLti151 [A, N, C, RC], bLti170 [A, N, C, RC]	H21	Ceará/Northeastern Brazil	Tadeu Gomes de Oliveira
bLti118 [A, N, C, RC], bLti157 [A, N, C*, RC], bLti164 [A, N, C, RC], bLti222 [A, N, C, RC]	H22	Maranhão - Piauí/ Northeastern Brazil	Tadeu Gomes de Oliveira
bLti156 [A, N, C, RC], bLti219 [A, N, C, RC]	H23	Pernambuco - Piauí/ Northeastern Brazil	Tadeu Gomes de Oliveira/ Zoológico de Recife
bLti147 [A, N, C, RC], bLti155 [A, N, C, RC]	H24	Maranhão/ Northeastern Brazil	Lígia Tchaicka/Tadeu Gomes de Oliveira
bLti85 [A, N, C, RC]	H25	Unkown origin	NCI-USA
bLti24 [A, N, C, RC]	H26	Goiás/ Central Brazil	Zoológico de Goiânia (NCI-USA)
bLti28 [A, N, C, RC]	H27	Distrito Federal/ Central Brazil	Zoológico de Brasília (NCI-USA)
bLco20 [A, N, C, RC], bLco27 [A, N, C, RC]	H28	Rio Grande do Sul/Southern Brazil	Felipe Peters
bLco21 [A, N, C, RC]	H29	Rio Grande do Sul/Southern Brazil	Fundação Zoobotânica do Rio Grande do Sul
bLco29 [A, N, C, RC]	H30	Rio Grande do Sul/Southern Brazil	LBGM – PUCRS
bLco32 [A, N, C, RC]	H31	Canelones/Uruguay	Museu Nacional de História Natural de Montevidéu
bLco17 [A, N, C, RC], bLco26 [A, N, C*, RC]	H32	Rio Grande do Sul/Southern Brazil	Jan Mähler/Felipe Peters
bLco28 [A, N, C, RC]	H33	Rio Grande do Sul/Southern Brazil	LBGM – PUCRS
bLco06 [A, N, C, RC]	H34	Soriano/Uruguay	Parque Zoológico de Mercedes (NCI-USA)
bLco16 [A, N, C, RC]	H35	Rio Grande do Sul/Southern Brazil	Glayson Bencke/Jorge Marinho
bLco11 [A, N, C, RC], bLco38 [A, N, C, RC]	H36	Rio Grande do Sul/Southern Brazil	Zoológico de Sapucaia do Sul/ Flavia P. Peter e Virgiane Knorr
Lco23 [A, N, C, RC]	H37	La Paz/Bolivia	Zoológico de La Paz (NCI-USA)
bLco04 [A, N, C, RC]	H38	Mendoza/Argentina	Parque Zoológico de Mendoza (NCI-USA)
bLco05 [A, N, C, RC]	H39	Córdoba/Argentina	Parque Zoológico de Córdoba (NCI-USA)
Lco09 [A, N, C, RC]	H40	Soriano/Uruguay	Parque Zoológico de Mercedes (NCI-USA)
bLco31 [A, N, C*, RC]	H41	Mato Grosso/Central Brazil	Carolina C. Cheida

Samples	Haplotype	Geographic origin	Institution/contact
bLco14 [A, N, RC]	H42	Catamarca/Argentina	Lúcia Soler
bLco30 [A, N, C*, RC]	H43	Goiás/ Central Brazil	Guilherme Miranda/Gustavo Chemale
bLti175 [A, N, C*, RC]	H44	Ceará/Northeastern Brazil	Tadeu Gomes de Oliveira
bLti152 [A, N, C*, RC]	H45	Piauí/ Northeastern Brazil	Tadeu Gomes de Oliveira
Lco10 [A, N, C*, RC]	H46	5ª Região/Chile	Zoológico de Quilpue (NCI-USA)
Lco12 [A, N, RC]	H47	5ª Região/Chile	Mina el Soldado (NCI-USA)
Lco11 [A, N, C*, RC]	H48	5ª Região/Chile	Zoológico de Quilpue (NCI-USA)
Lco26 [A, N, C*, RC]	H49	Iquique /Chile	SAG (NCI-USA)
B1 **	H50	Tacna - Puno/Peru	Cossíos *et al.* (2009)
B4 **	H51	Catamarca - Salta/Argentina	Cossíos *et al.* (2009)
B2**	H52	Potosi/Bolivia	Cossíos *et al.* (2009)
B3**	H53	Jujuy/Argentina	Cossíos *et al.* (2009)
A1**	H54	Tacna - Puno/Peru	Cossíos *et al.* (2009)
A3**	H55	La Paz - Onuro/Bolivia	Cossíos *et al.* (2009)
A4**	H56	Yauyos - Canchayllo/Peru	Cossíos *et al.* (2009)
A19**	H57	Junin National Reserve/Peru	Cossíos *et al.* (2009)
A16**	H58	Lambayeque/Peru	Cossíos *et al.* (2009)
D10**	H59	Pilcaniyeu/Argentina	Cossíos *et al.* (2009)
D1**	H60	Buenos Aires/Argentina	Cossíos *et al.* (2009)
D6**	H61	San Juan - Mendoza/Argentina	Cossíos *et al.* (2009)
C3**	H62	Potosi/Bolivia	Cossíos *et al.* (2009)
C11**	H63	Jujuy/Argentina	Cossíos *et al.* (2009)
C12**	H64	Catamarca - Salta/Argentina	Cossíos *et al.* (2009)
C6**	H65	Potosi/Bolivia	Cossíos *et al.* (2009)
C5**	H66	Potosi/Bolivia	Cossíos *et al.* (2009)
C9**	H67	Jujuy/Argentina	Cossíos *et al.* (2009)
D8**	H68	Catamarca - Salta/Argentina	Cossíos *et al.* (2009)
bLti72 [A, N, C, RC]	H69	Mato Grosso do Sul/Central Brazil	Zoológico de Catanduva

Samples	Haplotype	Geographic origin	Institution/contact
bLti96 [A, N, C, RC]	H70	Goiás/ Central Brazil	Flávio Rodrigues
bLti209 [A, N, C, RC]	H71	Distrito Federal/ Central Brazil	Criadouro de Morro Reuter
bLwi32 [A, N, C, RC]	H72	São Paulo - Brazil	Zoológico de Tapiraí
bLpa72 [A, N, C*, RC]	H73	São Paulo/Southeastern Brazil	Zoológico de São Bernardo do Campo

Note:

** Shorter segments from Cossíos et al. (2009), including *ATP8* (133bp), *ND5* (250bp) e Control Region (171pb).
