# Supplementary material for: Phylogeographic analyses of the pampas cat (Leopardus colocola; Carnivora, Felidae) reveal a complex demographic history
Source: Genet Mol Biol. 2018;41(1 Suppl 1):273–87. doi: 10.1590/1678-4685-GMB-2017-0079 (PMC5913729; doi:10.1590/1678-4685-GMB-2017-0079)
Supplement: Supplementary file 2 [file 1415-4757-GMB-41-01-2017-0079-s002.pdf]

Supplementary Material to "Phylogeographic analyses of the pampas cat (*Leopardus colocola*; Carnivora, Felidae) reveal a complex demographic history"

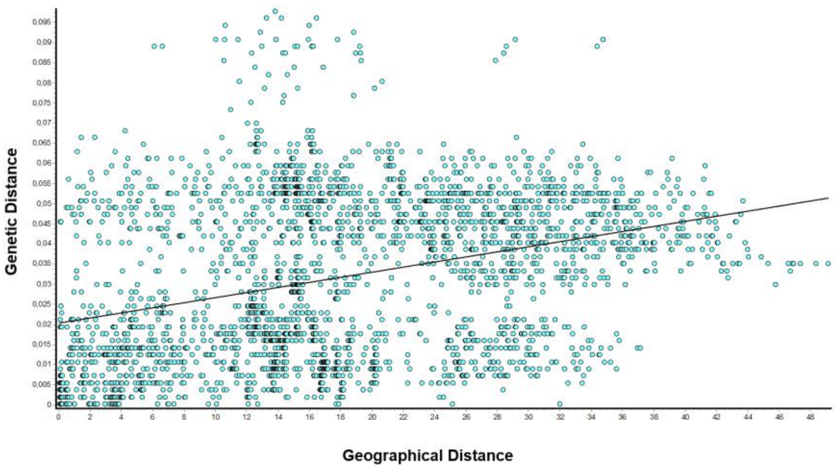

A

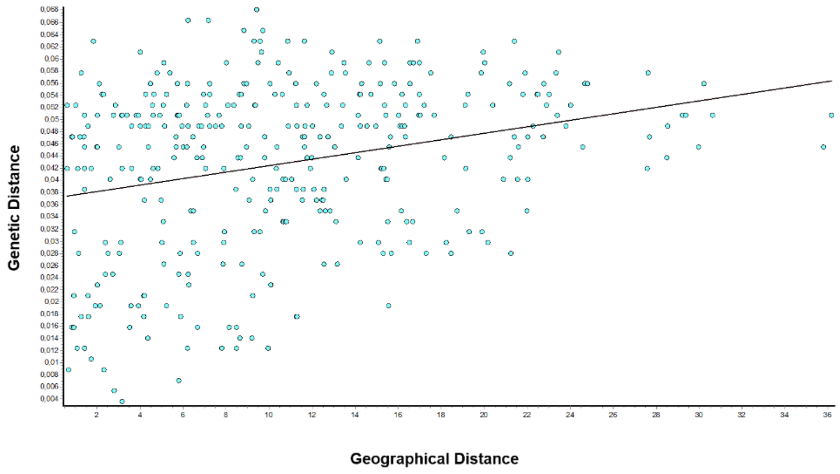

B

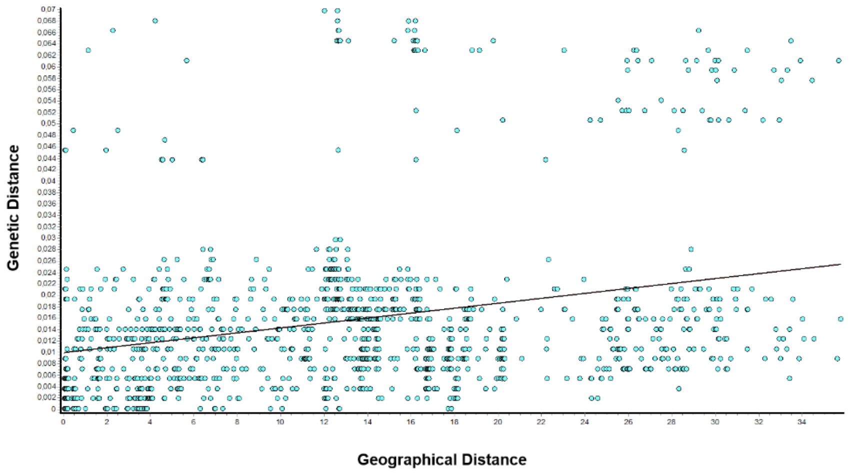

C

Figure S1 - Correlation between genetic and geographic distance for *L. colocolo* and *L. tigrinus* haplotypes, estimated with the concatenated data set (all sites with missing data were excluded). **A)** The entire data set B (DSB). **B)** Samples from western South America. **C)** Samples from eastern South America.
