# Supplementary material for: Phylogeographic analyses of the pampas cat (Leopardus colocola; Carnivora, Felidae) reveal a complex demographic history
Source: Genet Mol Biol. 2018;41(1 Suppl 1):273–87. doi: 10.1590/1678-4685-GMB-2017-0079 (PMC5913729; doi:10.1590/1678-4685-GMB-2017-0079)
Supplement: Supplementary file 3 [file 1415-4757-GMB-41-01-2017-0079-s003.pdf]

Supplementary Material to “Phylogeographic analyses of the pampas cat (*Leopardus colocola*; Carnivora, Felidae) reveal a complex demographic history”

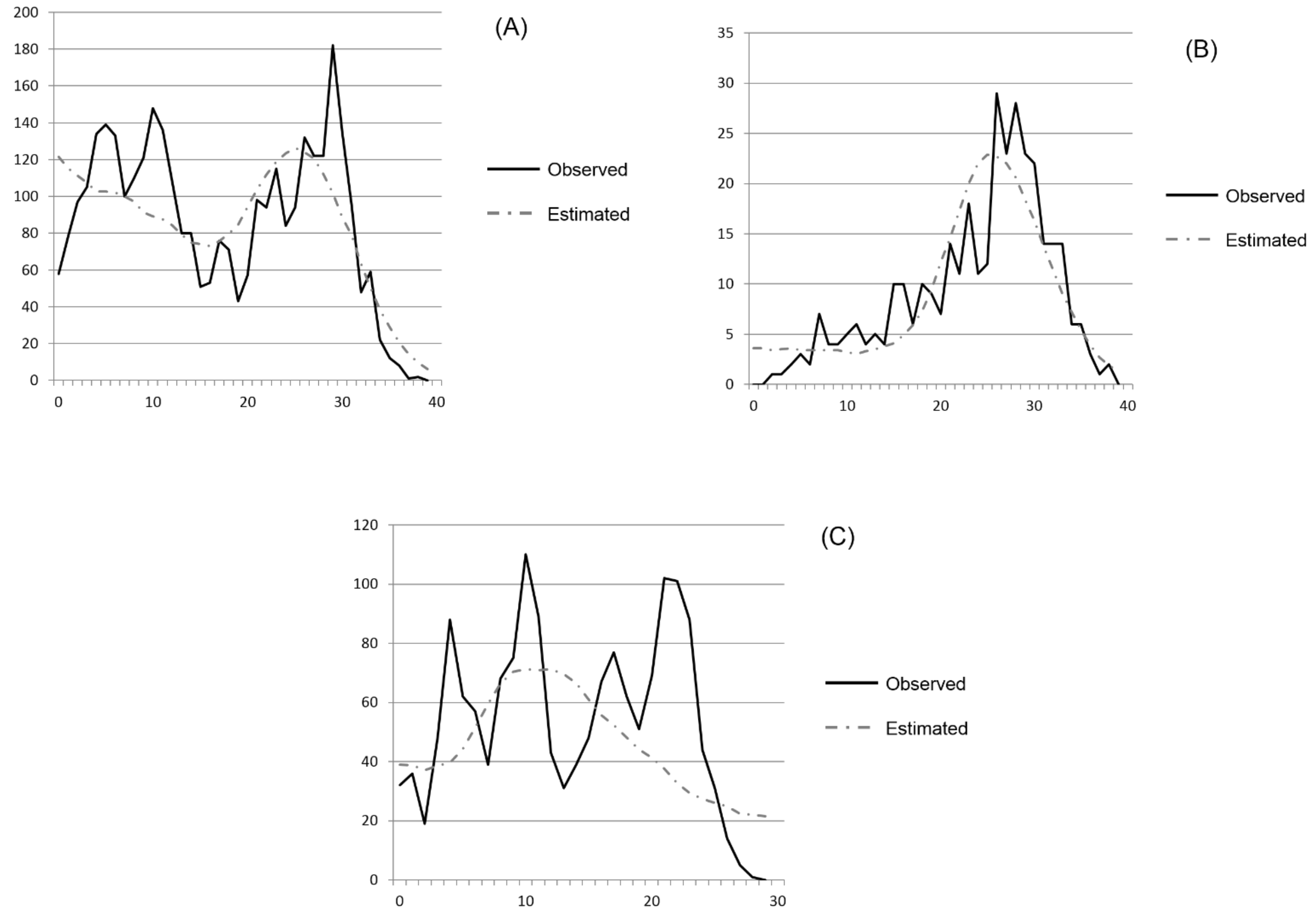

Figure S2 – Analysis of the pairwise nucleotide differences distribution (Mismatch Distribution) of mtDNA haplotypes assessed in this study. The solid line indicates the observed frequency of pairwise differences between haplotypes, while the dashed line represents the expected frequency under a sudden expansion model. A) Graph of the entire set of samples. B) Graph including only samples from western South America C) Graph for samples from eastern South America.
